# Toward the Industrial Application of Aluminum-Based Nanocomposite Material: A Study of Zn-Plating Process in Circuit Breaker Application

**DOI:** 10.3390/nano12193535

**Published:** 2022-10-10

**Authors:** Emmanuel Segura-Cárdenas, Nicolás A. Ulloa-Castillo, Roberto Hernández-Maya, Oscar Martínez-Romero, Alex Elías-Zúñiga

**Affiliations:** 1 School of Engineering and Sciences, Tecnologico de Monterrey, Av. Eugenio Garza Sada Sur 2501, Monterrey 64849, Nuevo León, Mexico; 2 Center for Innovation in Design and Technology, Department of Mechanical Engineering and Advanced Materials, School of Engineering and Sciences, Tecnologico de Monterrey, Av. Eugenio Garza Sada Sur 2501, Monterrey 64849, Nuevo León, Mexico; 3 Siemens, Research and Development Department, Libramiento Arco Vial Poniente Km 4.2, Santa Catarina 66350, Nuevo León, Mexico; 4 Department of Mechanical Engineering and Advanced Materials, Institute of Advanced Materials for Sustainable Manufacturing, Tecnologico de Monterrey, Av. Eugenio Garza Sada Sur 2501, Monterrey 64849, Nuevo León, Mexico

**Keywords:** multiwall carbon nanotubes, coating, mechanical zinc plating, coated aluminum nanocomposite circuit breaker part, molded case circuit breaker device

## Abstract

This article explores the industrial application of an Al-based nanocomposite reinforced with 0.5 wt.% of multiwalled carbon nanotubes with a Zn mechanical plating applied to fulfill the field requirements of electrical devices. The performance of electric devices made from this nanocomposite material and with a Zn plating was compared with that of MCCB devices using a normal Cu compound. MCCB devices with the Al-based nanocomposites compound showed a better performance, with less heat generated due to a flow of electrical charge passing through the device. The presence of MWCNTs in the Al nanocomposite dissipates heat, maintaining a stable electrical resistance in the MCCB, in contrast to what happens with Cu compound, which increases its electrical resistance as the temperature in the device increases.

## 1. Introduction

In highly conductive pure metals, such as silver (Ag), copper (Cu), and aluminum (Al), the resistance and electrical conductivity are the two most important properties for electrical conductivity applications. In addition, the development of metal-based nanocomposites of such materials are of great relevance to improve electrical behavior. In this sense, the main challenge lies in exploring experimental routes capable of preserving the intrinsic properties of the nanocomponents to develop a nanocomposite material with enhanced properties [1,2,3].

Recently, Al-based nanocomposites have been developed with improved electrical properties. Carbon nanotubes (CNTs) have emerged as an excellent reinforcement to design novel Al-based CNT nanocomposites [4,5,6,7] due to their exceptional electrical, mechanical, and thermal properties [8,9,10] that improve the strength and electrical conductivity of metals [11]. There are several strategies to achieve functional metal-based nanocomposite materials such as chemical modification assisted by using molten salts [12,13] and mechanical adhesion [14]. Both are able to improve the chemical interaction between the metal matrix and the reinforcing materials, avoiding agglomerations. Despite such strategies being ideal to achieve optimal CNT dispersion, there are cost limitations to scale the developed material into mass production. In this sense, the ball-milling process helps to achieve homogenous dispersion of CNTs during the preparation of nanocomposite materials, so that they can be used in mass production.

On the other hand, surface treatments and enhancement of properties of the metal matrix, by applying protective coatings, has played a significant role in the fight against corrosion, which affects the material’s electrical performance. Coatings offer protective barrier between the corrosive environment and the substrate [15]. There are various types of coatings that range from nickel (Ni) to copper (Cu), zinc (Zn), and silver (Ag). Zn coatings have been successfully used over the past decades to mitigate the corrosion deterioration of mild steel due to their good anti-corrosion properties, ease of fabrication and affordability. Zn is more active than iron or steel and, thus, offers sacrificial protection to the base metal [16]. There are different coating deposition techniques [17,18,19]. Each method has its own advantages and disadvantages, depending on the desired properties and expectations. However, high-temperature coating methods tend to introduce hydrogen embrittlement [20]. Mechanical plating is a process that can be performed at room temperature. In this sense, the most important of all, the absence of electrolysis, removes at a stroke any danger from hydrogen embrittlement [20]. The mechanical plating is evaluated in terms of operation, application, cost, and product properties.

This article aims to investigate the influence that the Zn-plating process has on the electrical properties of industrial samples produced from an Al matrix reinforced with 0.5 wt.% concentration of multiwalled carbon nanotubes (MWCNTs), as described in [21,22,23]. The coated nanocomposite samples were used as contact plating material options for a molded case circuit breaker (MCCB) device to evaluate its electrical performance under engineering applications. The coated nanocomposite samples were subjected to experimental characterizations to evaluate the coating process, coated layer adhesion, and electrical properties. The tribological tests showed that the Zn coatings for the metallic nanocomposites could be considered as soft coatings due to the resulting surface morphology. However, the tribological properties of these Zn coatings are good enough to prevent corrosion on the produced Al-nanocomposite part, which could hinder the material’s electrical performance.

## 2. Materials and Methods

Industrial-grade MWCNTs (5–15 nm average diameter and 10–20 μm length) were purchased from Nanostructured & Amorphous Materials, Inc., Houston, TX, USA. Stearic acid and powdered zinc (with an average size of 5 µm) materials were acquired from Sigma Aldrich St. Louis, MO, USA. Aluminum powder (>99%) was purchased from Ampal Inc., Palmerton, PA, USA.

### 2.1. Preparation of Nanocomposites

The processing and preparation of the Al-based nanocomposite powder was carried out using attrition ball-milling equipment (Union Process Inc., Akron, OH, USA), following the same procedure described in [23], which addresses the industrial scaling up of Al-based nanocomposite materials. In this sense, we used the best experimental conditions to achieve both a homogenous dispersion of MWCNTs and the sintering consolidation to preserve electrical conductivity. Hence, the nanocomposite powder was dispersed into the Al matrix, adding 0.5 wt.% of MWCNTs, and stearic acid (0.5 wt.%) was used as a process control agent [24,25]. The milling process was performed using a rotation velocity of 215 rpm during 5 min. The obtained nanocomposite powders were uniaxially compressed in an electrical compound-shaped mold made from a steel tool and then sintered in a 12 in (0.304 m) controlled atmosphere-hardening furnace to obtain parts with 10 mm thickness.

### 2.2. Mechanical Plating of Zinc

**Figure 1:**
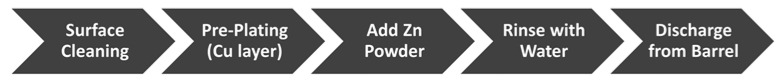
Mechanical plating procedure.

The condition of the surface is relevant to achieving a good plating process, so sintered Al-based nanocomposites were wet-chemical etched using a solution based on H_2_SO_4_ and H_2_O in a volume ratio of 3:1 for 10 min and rinsed with water in order to remove undesirable particles from the surface prior the plating process. The plating process was carried out through a mechanical plating system in which the sintered parts were placed into a rotating drum container, which was operated at 50 rpm for 10 min using glass beads with diameters ranging from 0.2 to 4 mm. Firstly, a Cu pre-plating was carried out through a solution based on copper powder, tin salt, and water. After the Cu pre-plating process, Zn powder was added to the container, and the plating process was finally completed by operating the system with the same experimental conditions previously described and following the ASTM 695B standard norm (Figure 1). Both Cu pre-plating and Zn-plating processes were performed on both sides (top and bottom) of the sintered Al-based parts. The dimensions of the parts were 6.45 × 4.78 cm, with 1.27 cm thickness, in a rectangular shape.

### 2.3. Surface Morphological Characterization

The surface morphological study of the Zn-plated parts was carried out using SEM equipment (ZEISS model EVO MA 25) which was operated at 20 kV acceleration voltage. The corresponding micrographs were taken using both secondary electrons (SE) and backscattered electrons (BSE) to validate the Zn-plating process. In the same way, an energy dispersive spectroscopic (EDS) analysis was used to explore the chemical composition and elemental mapping of the Al-based nanocomposite parts.

### 2.4. Microindentation Hardness Tests

The validation of the Zn-plating process was carried out through a micro indentation Daimler-Mercedes Rockwell-C adhesion test following the VDI 3198 standard norm, which consisted of applying a load of 150 kg using an indenter made of diamond with a conical shape to explore if the plating layer was detached from the surface of the nanocomposite parts.

### 2.5. Tribological Characterization

Friction testing was carried out with tribometer equipment (UMT TriboLab-Bruker) using the pin-on disk configuration according to the ASTM G99-05 (standard test method for wear tests with a pin-on disk machine), which is a standard method for obtaining valid data. The contact ball was made of Al_2_O_3_ with a diameter size of 6 mm. The normal load, rotating diameter, frequency, and testing time were 10 N, 10 mm, 0.5 Hz, and 300 min, respectively. During testing, the friction coefficient was continuously recorded.

### 2.6. Electrical Conductivity of the Zn-Plated Nanocomposites

The electrical conductivity measurements were carried out on four different samples of the coated nanocomposites using the four-wire method, whose setup configuration consisted of DC system power supply equipment (model SPM10, purchased from Fisher, Monterrey, Nuevo León, Mexico). The tests were performed in accordance with the ASTM E 1004 and DIN EN 2004-1 standard norms. The electrical conductivity, σ (S/m), was determined using the expression σ=1/𝜌 where *𝜌* is the electrical resistivity in Ω m.

### 2.7. Electrical and Thermal Test Configuration of the Zn-Plated Parts Adapted to a Molded Case Circuit Breaker

**Figure 2:**
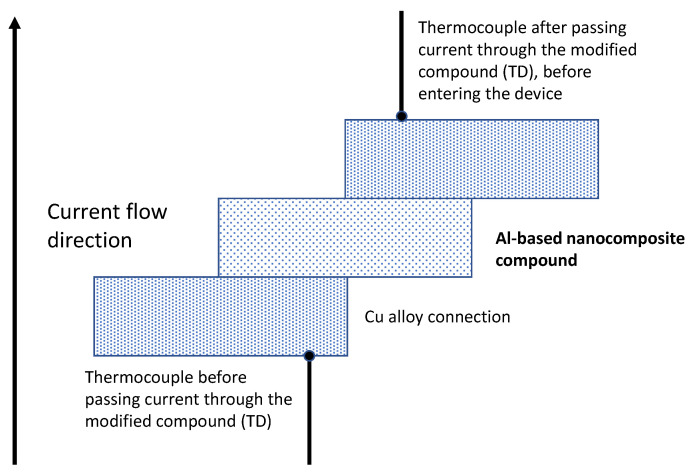
Diagram showing the place of the thermocouples for the home-made test of current at 80% and 100% of the nominal current of the MCCB device.

A molded case circuit breaker (MCCB) is a device for electrical protection that can be used for wide range of voltages, which could be ramped up to 2500 amperes, with a trip unit, which is commonly adjustable. The molded case circuit breaker was manufactured by the Siemens company to work with a capacity of 1200 amps. The electrical performance testing was carried out using both 80% and 100% of the nominal current capacity. The experimental configuration for the electrical tests of the parts in the molded case circuit breaker is shown in Figure 2.

Additionally, we modified the experimental configuration to test the thermal behavior of the Zn-plated parts during the electrical test performance of the molded case circuit breaker. In this case, type J thermocouples (Omega, Spain) were adapted to the terminals (input/output) of the circuit breaker to monitor the temperature as shown Figure 1.

## 3. Results and Discussion

### 3.1. Morphological Zinc Coating Samples

Ag is typically used as a coating to protect metallic surfaces from corrosion phenomena in electrical applications. However, the processing cost is commonly expensive [26]. The authors have identified that a suitable replacement can be Zn since it was demonstrated that it can be used to avoid corrosion phenomena, as well as having a lower processing cost making it suitable for large-scale production of parts. The central role of the Zn coating is to prevent hydrogen embrittlement of the surface, a common issue observed in mechanical plating processes [27,28].

**Figure 3:**
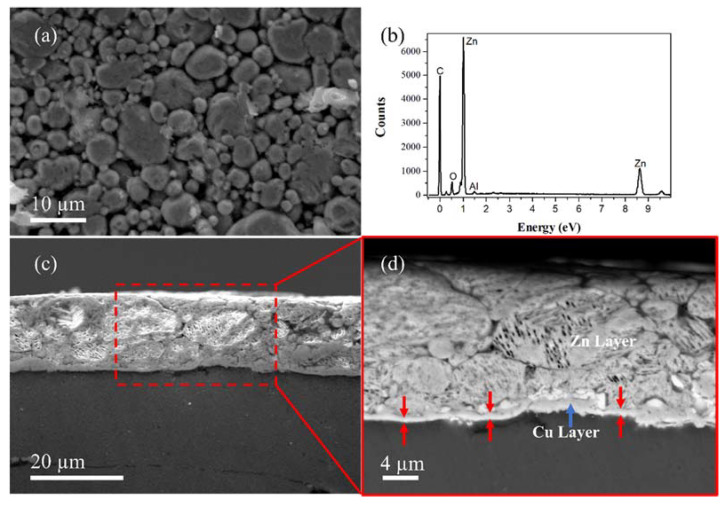
(**a**) SE-SEM of coating surface finishing. (**b**) EDS-SEM analysis of coating. (**c**) Cross-section of coated nanocomposite and coating thickness. (**d**) Interface layer between the Al-based nanocomposite surface and the Zn-coated layer.

Figure 3a shows the top view of the coated layer in which it is possible to note the presence of some particles and agglomerations that were not consolidated properly, revealing that the industrial procedure for mechanical plating of the nanocomposite parts can be improved. The thickness of the coated layer was measured by taking cross-sectional SEM micrographs (Figure 3c), obtaining values ranging from 12 µm to 18 µm. Such variations could be due mainly to the low particle consolidation during the plating process. Figure 3d shows a zoom-in view of the cross-section area revealing two main features, the Cu pre-plating of a thin layer between the nanocomposite and the Zn layer.

The Cu pre-plating layer was mechanical coated to promote a better interaction between the Al-based surface and the Zn-plating layer. In this sense, it is of relevance to note that the plating conditions were optimal for the Cu layer rather than the Zn layer, which exhibited large zones with cracks and pores.

### 3.2. Microindentation Analysis

**Figure 4:**
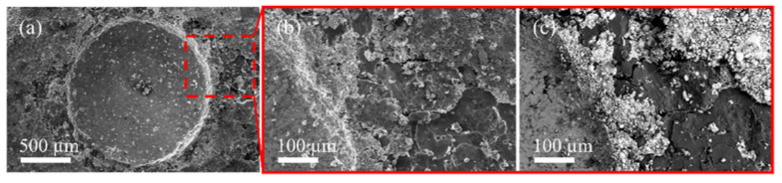
(**a**) SE-SEM micrograph of the indentation print on coated nanocomposite. Zoom-in view of the delamination of the material around the trace of the Zn coating in (**b**) SE-SEM and (**c**) BSE-SEM images.

The condition of the Zn-plating layer was observed by SEM micrographs, and the quality of the coating was also tested through a microindentation test. Figure 4a shows the indentation footprint of the coated nanocomposite. SEM micrographs of coated nanocomposite provide practical and applicable information as a destructive quality test for coated compounds according to the VDI 3198 standard.

A notable number of chipped areas around the footprint were observed and these were related to the nanocomposite coating peeling off. Figure 4b shows a zoom-in view of the chipped areas analyzed by SE-SEM. One can see delamination and cracks around the footprint. The latter was corroborated using BSE-SEM (Figure 4c). The micrograph shows a contrast of intensities that indicates the presence of two different materials. The dark zone is related to the presence of Al that has been revealed after the detachment of the coating layer. The brighter zone is related to the Zn traces after the indentation process.

As expected, detachment of the Zn layer was observed, which corroborates the previously discussed results of the micrographs obtained by SEM measurements. Since the thickness of the pre-plating layer was around 670 nm, it was difficult to distinguish if the coating had been detached from the surface.

### 3.3. Tribology Analysis

**Figure 5:**
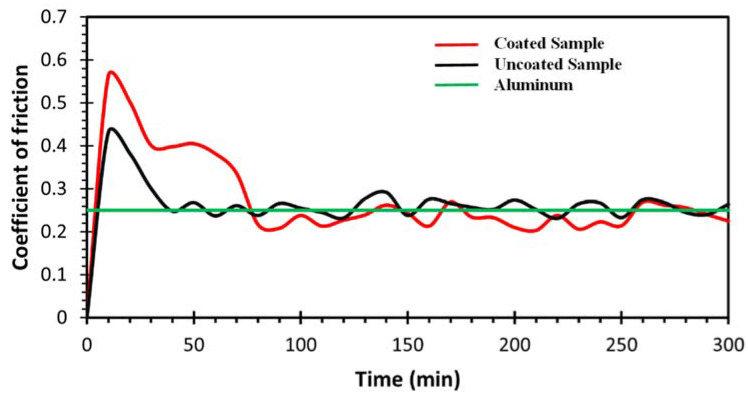
Coefficient of friction (COF) of aluminum, uncoated, and coated nanocomposite samples.

Figure 5 shows the coefficient of friction (COF) as a function of the time traveled by the ball on aluminum, uncoated nanocomposite, and coated samples. Aluminum showed a COF value of 0.25 when its surface was subjected to friction tests using an alumina ball [29,30]. It was observed that the uncoated sample reached a maximum COF value of 0.42 in the first 30 min of the ball’s travel. For times greater than 40 min, the COF value oscillated around 0.25. In the case of coated nanocomposite, the test showed a similar trend, followed by the uncoated sample. In the first few minutes, the coated sample reached a maximum of 0.58. In the following 60 min, the COF value decreased to an average value of 0.40. After 90 min, the coefficient of friction value fluctuated around 0.25. According to the results obtained, the Zn coating showed little adherence to the surface of the nanocomposite, since the samples showed similarly close values at the end of the test. The latter is indicative that the Zn material was detached from the surface of the nanocomposite.

**Figure 6:**
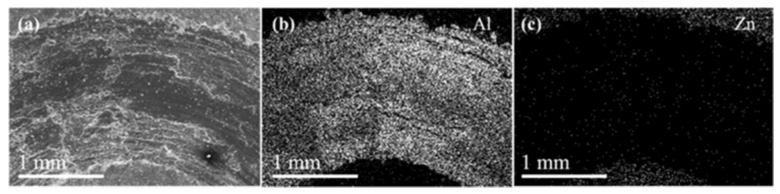
SE-SEM micrograph (**a**) coated nanocomposite sample wear. Elemental mapping of the coating after wear test: (**b**) Al and (**c**) Zn.

Figure 6 shows the SEM images of coated nanocomposite sample wear. The wear test marks indicate that there was a material detachment in the area tested and in the surroundings of the printed mark. The EDS-SEM elemental mapping performed on the tested surface revealed that the element present in greater abundance was aluminum (Figure 6b). This indicates that the Zn coating flaked off during the test (Figure 6c). SEM micrographs revealed delamination, plow marks, and grooves in the slip direction on the worn surfaces (Figure 6a). It was also observed that the imprint in the wear zone had a width of around 1.84 mm. The applied load of 20 N on the surface of coated nanocomposite showed that the adhesion of the coating was weak due to the detachment of Zn from the sample surface.

The EDS elemental mapping analysis, shown in Figure 6c, indicates that both plated layers (Cu and Zn, respectively) were almost completely detached exposing the Al surface after the wear test, as observed for the Al mapping in Figure 6b. The latter was also corroborated by the pin-on disk test (Figure 4), where the COF values were similar for plated and non-plated parts.

### 3.4. Electrical Performance of Al-Based Nanocomposite in a Molded Case Circuit Breaker

The electrical conductivity of the different coated samples was measured to evaluate the electrical performance. The values obtained from the different coated samples S2 and S3 were compared with those of the uncoated samples I1 and I2. The results are summarized in Table 1. Note in Table 1 that the average value obtained in all coated samples was around 24.6 MS/m, which corresponds to 41.57% in the conversion of the International Annealed Copper Standard (IACS). The electrical conductivity values found in the uncoated samples showed a similar value to the coated samples. As previously discussed in [21,23], the well-dispersed MWCNTs allowed formation of an effective network in the Al matrix, promoting an improvement of the electrical conductivity [31,32].

**Figure 7:**
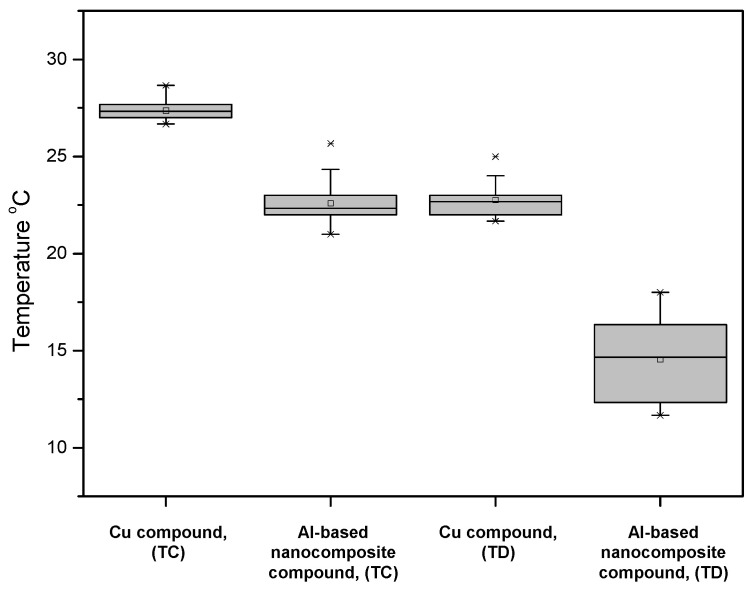
MCCB device temperature operating at 80% rated current monitored at terminals TC and TD of the device configuration.

An experimental configuration was used to measure the samples’ thermal performance. Figure 7 shows the results obtained from the performance of the circuit breaker device subjected to 80% of its nominal current. The temperature value (23 °C) of the Al-based nanocomposite compound was lower than the temperature value (27 °C) of the Cu compound at the TC terminal that was made from sintered Cu alloy CDA 110, with a silver-plated surface finish. The temperatures recorded at the TD terminal showed a behavior like that reported at the TC terminal. The temperature of the nanocomposite sample was about 8 °C lower than that of the Cu compound.

These tests reveal that the nanocomposite compound showed a better performance in comparison to the Cu compound in terms of the current flow. The behavior exhibited by the nanocomposite plated compound was due to the MWCNTs as reinforcement in the composite material, where the MWCNTs formed an interconnecting network with Al- milled particles and a percolation threshold phenome, discussed previously by the authors in [19]. In terms of the thermal conductivity, the MWCNTs as reinforcement in Al- matrix materials maintained their exceptional properties, including high thermal conductivity, and after milling and due to the reached dispersion of the reinforcement, the performance was better in comparison with that of the normal Cu compound [33]. Even if the Zn-plating layer was not optimal and it was delaminated in a short time; as observed in Figure 5, the electrical performance was preserved and protected the surface from corrosion phenomena. The latter provided information about the tribological properties of the nanocomposite and its use as a contact in a molded case circuit breaker (MCCB) for electrical applications.

**Figure 8:**
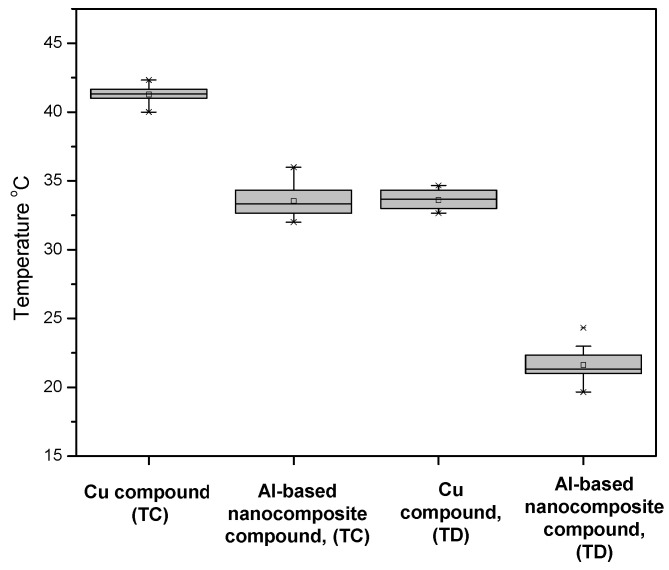
MCCB device temperature operating at 100% rated current monitored at terminals TC and TD of device configuration.

Figure 8 shows the results obtained from the performance of the MCCB device subjected to 100% of its nominal current. The temperature value (33 °C) of the nanocomposite compound was lower than the temperature value (41 °C) of the Cu compound at the TC terminal, before the current entered the composite material. The temperatures recorded at TD terminal, after a current was passed through the composite material, showed a behavior like that reported at the TC terminal. The temperature of the nanocomposite compound was about 12 °C lower than that of the Cu compound. The nominal current supplied to the device was 20% higher compared to that in the first study. This showed an increase of the heat generated when current flowed through the device. However, the behavior of the two different compounds used during the test presented a performance similar to that reported for a nominal current of 80%. This demonstrates that Al-based nanocomposite compounds had better performance in both tests.

The material’s thermal dissipation was due to the presence of carbon nanotubes, which were located around the aluminum grains and, as mentioned above, were distributed in such a way that they created a network within the material, allowing increasing thermal dissipation [34,35]. This finding is relevant since the electrical conductivity of the device was not reduced due to the heating of the material during the device operation. This behavior was different in the Cu compound since an increase in its electrical resistance caused an increase in the device temperature [36].

It is evident that the coating process on the aluminum-composite samples can control the electrical resistance and thermal properties of internal circuit breaker parts during their manufacturing processes. Our results validate that these properties remained almost the same after the Zn-plating process, and thus, one can conclude that Zn plating’s main function was to protect the circuit breaker during production. Furthermore, the Zn coating protected the coated part from corrosive environments and resulted in a technical advantage for components subjected to corrosive environments.

## 4. Conclusions

The coating process for metallic aluminum compounds was carried out through mechanical plating, which was identified as a conventional industrial mass production process for MCCB devices. The quality of the coating was evaluated through tribological tests, which showed that the parameters used during mechanical plating can be im-proved to obtain homogeneity in the desired film. Furthermore, the electrical properties of Zn-plated nanocomposite samples were evaluated, and the results obtained indicate that the coating deposited keeps the electrical conductivity close to 41% of the IACS.

In summary, we can conclude that:

1. The Cu pre-plating layer showed good homogeneous distribution on the surface of the aluminum nanocomposite, indicating an excellent interface between the nanocomposite materials and copper. However, the interface between Cu and the Zn coating had weak interaction between the materials due to the presence of semi-molten particles that caused pores formation in the coating layer.
2. The indentations of the tribological tests showed that the Zn coatings for the metallic nanocomposites should be considered as smooth coatings since the collected coefficient of friction values for both the coated and uncoated samples were similar.
3. The identification of plating process parameters plays a key role in obtaining good quality coating. Zn coatings can be used on Al nanocomposite materials to mitigate corrosion deterioration without hindering heat dissipation, while maintaining a stable electrical resistance in the MCCB, contrary to what happens with Cu, which in-creases its electrical resistance as the temperature in the device increases.
4. The developed aluminum-based nanocomposite compounds follow the RoHS compliance guide, showing good performance under the MCCB device operation conditions.
5. The aluminum-based nanocomposite material with Zn as coating exhibited improved properties in the home-made engineered test that was based on the UL 489 standard for electrical devices, against the typical used material, with a final cost reduction in the manufacturing process for the MCCB process.

## Figures and Tables

**Table 1:** Electrical conductivity values, calculated from the electrical resistivity, and their corresponding conversion to (IACS%) values of coated Al-MWCNT nanocomposite.

Sample	Description Sample	Conductivity (MS/m)	% IACS
I1	uncoated	24	41.7
I2	uncoated	24	41.7
S2	zinc coated	24.6	42.57
S3	zinc coated	24.6	41.57
